# Knowledge of cervical cancer prevention and treatment, and willingness to receive HPV vaccination among college students in China

**DOI:** 10.1186/s12889-022-14718-0

**Published:** 2022-12-05

**Authors:** Fengzhi Zhang, Manman Li, Xiaoxue Li, Hua Bai, Jinling Gao, Hua Liu

**Affiliations:** 1 Department of Gynecology, The Third Affiliated Hospital of Zhengzhou University, 7 Front Kangfu Street, Zhengzhou, 450052 Henan Province China; 2 School of Nursing and Health, Zhengzhou University, Zhengzhou, China; 3 School of Foreign Languages, Zhongyuan Institute of Science and Technology, Zhengzhou, China

**Keywords:** Cervical cancer, HPV vaccine, Willingness to vaccination, College students

## Abstract

**Background:**

Cervical cancer is the fourth most common cancer in women. Up to 99% of cervical cancer cases are associated with high-risk human papillomavirus (HPV). Sexual behavior is a direct risk factor for HPV infection, and sexually active college students, therefore, receive attention for HPV vaccination. At present, most Chinese studies lack of in-depth research on influencing factors, and are limited to cervical cancer, HPV, or HPV vaccine, without comprehensive consideration. This study investigated Chinese college students’ cervical cancer prevention and treatment knowledge level, and explored the influencing factors, and understood their willingness to receive HPV vaccination. The findings of this study will lay a foundation for promoting the early screening of cervical cancer and vaccination process.

**Methods:**

A total of 800 college students from four universities in Zhengzhou, China were selected by multistage random sampling method. A self-administered questionnaire on the knowledge of cervical cancer prevention and treatment, and willingness to receive HPV vaccination was carried out. A logistic regression model was conducted to analyze factors influencing knowledge of cervical cancer prevention and treatment among college students.

**Results:**

Up to 87.9% of college students said they had heard of cervical cancer. The proportion of college students with good knowledge of cervical cancer prevention and treatment was 46.7%. Logistic regression showed that gender, major, grade, level of education, the father's level of education, premarital sex attitude, and mother cervical cancer screening participation had a significant influence on cervical cancer prevention and treatment knowledge level (*P* < 0.05). In addition, 589 (74.0%) of college students had heard of HPV vaccine, and 92.8% of college students said they were willing to get vaccinated or recommended that their relatives and friends get vaccinated.

**Conclusions:**

The knowledge level of cervical cancer prevention and treatment knowledge among college students in Zhengzhou is low. Many of them had poor knowledge about HPV vaccine, but their willingness to vaccination is high. Various health education modes should be carried out for people with different characteristics, to improve their knowledge of cervical cancer prevention and promote the vaccination process.

## Introduction

Cervical cancer is the fourth most common cancer in women. More than 310 thousand women die of cervical cancer every year worldwide, and the age of onset tends to be younger, which brings a heavy economic burden to society and families. Accelerating the elimination of cervical cancer has become a global public health priority [1, 2]. Up to 99% of cervical cancer cases are associated with high-risk human papillomavirus (HPV). Despite most HPV infections resolving spontaneously without causing any symptoms, persistent infection can lead to cervical cancer in women [3]. HPV vaccine can reduce the incidence of cervical cancer by approximately 70%, in addition to preventing other diseases such as anal cancer, genital warts, and oropharyngeal cancer [4, 5]. The World Health Organization (WHO) points out that in order to improve the vaccination rate, the vaccination should be carried out at the same time as other vaccines of the same age, or based on the school and adolescent health services [3]. Many countries have been included in the national immunization program, and the HPV vaccination program has tended to be perfect. However, the current HPV vaccine in China belongs to the non-immunization program vaccine, the domestic vaccine awareness and vaccination is not mature, the age-appropriate girls HPV vaccine coverage rate is less than 1% [3, 6]. Sexual behavior is a direct risk factor for HPV infection, and sexually active college students, therefore, receive attention for HPV vaccination [7]. As one of the target groups, college students have a broad research prospect. At present, most Chinese studies lack of in-depth research on influencing factors, and are limited to cervical cancer, HPV, or HPV vaccine, without comprehensive consideration.

The objectives of the present study were to investigate 1) knowledge level of cervical cancer prevention, 2) the factors influencing the knowledge of cervical cancer prevention and treatment, and 3) willingness to receive HPV vaccination among college students in Zhengzhou, Henan Province, China. The results of the study could lay a foundation for the prevention of cervical cancer.

## Methods

### Study population and eligibility criteria

Students who gave informed consent and voluntary participation were included from participation. The exclusion criteria were students who 1) were confirmed severe physical illness; 2) were confirmed severe mental disorders; 3) couldn’t communicate effectively.

### Measurement

According to relevant studies published in China and other countries [7–9], we designed the questionnaire on the basis of experts’ consultations. A pre-survey was conducted before the formal survey, and the final questionnaire was obtained after revision. The content of the questionnaire included the demographic characteristics of the participants, their knowledge of cervical cancer prevention and treatment, and their willingness to vaccination, etc. The demographic information included age, major, class, place of household registration, level of education, mean monthly consumption, and parents’ level of education. Knowledge of cervical cancer prevention and treatment included knowledge of cervical cancer, HPV, and HPV vaccine. The questionnaire was based on three expert consultations. In the first expert consultation, experts suggested adding HPV-related knowledge and other items, and revising some unclear items, such as: "Do your family members or friends with cancer?" changed it to "Do you have family members or friends with current or history of cancer?". For the second time, experts suggested changing the multiple-choice questions to true-or-false questions and setting reverse scoring crtitera. In the third expert consultation, there was no objection to the questionnaire. The final questionnaire on the knowledge of cervical cancer prevention and treatment contained 35 items (five multiple-choice questions and 30 true-or-false questions). The scoring method was: one point for each correct answer and 0 point for each incorrect or no answer. The total score ranged from 0 to 35 points. The *Cronbach’s α* coefficient of the questionnaire on the knowledge of cervical cancer prevention and treatment in the pre-survey was 0.907; the *Cronbach’s α* coefficient of the post-test questionnaire was 0.953, indicating satisfactory internal consistency.

### Data collection

Three research assistants were hired for this study to collect data, including two graduate students and one nurse practitioner. Before the formal investigation, they were trained on study protocol and explanation of questionnaire items by the principal researcher. Finally, they took a test on the study protocol and questionnaire content with a passing score of 90 or above. All three research assistants passed the exam and were allowed to participate in this study.

From October to December 2020, a total of 800 college students aged 17–23 years old from four universities were recruited in Zhengzhou, Henan Province, China. A stratified cluster random sampling method was used according to the students’ major and class. In each university, one class was selected at random according to the grade level. The study took place after class. The research assistants explained the purpose and significance of the study to the participants. All participants received informed consent before enrolling in the study and were guaranteed voluntary participation, withdrawal, study risks, confidentiality, and secured data storage.

### Statistical analysis

The data were analyzed using SPSS v22.0 software (IBM Corp., Armonk, NY, USA). Measurement data were expressed as means ± standard deviation ($\overline{X }$ ± S); count data were expressed as frequency and percentage (%). The scores of cervical cancer prevention and treatment knowledge showed a skewed distribution, with a median of 13. A score ≥ 13 was defined as good knowledge of cervical cancer prevention and treatment and assigned a value of 0. A score < 13 was defined as poor knowledge of cervical cancer prevention and treatment and assigned a value of 1. To explore the factors influencing the knowledge of cervical cancer prevention and treatment among college students in Zhengzhou, Mann–Whitney U testing was used for used for univariate analysis. Logistic regression was used for multiple covariates to explore the factors influencing the knowledge of cervical cancer prevention and treatment among college students. A *P* value < 0.05 was considered statistical significance.

## Results

### Demographic characteristics of the participants

A total of 800 questionnaires were sent out and 796 valid ones were collected, with a valid rate of 99.5%. The mean age of the participants was 19.71 (SD 1.25). Most (87.9%) participants indicated that they had heard of cervical cancer. The proportion of college students with good knowledge of cervical cancer prevention and treatment was 46.7%. Seven hundred forty-three (93.3%) participants were willing to learn about the knowledge of cervical cancer prevention and treatment. The demographic characteristics of the participants were summarized in Table 1.

**Table 1:** Demographic characteristics of the participants (*n* = 796)

Variable	Group	Frequency (n)	Percent (%)
Gender	Male	117	14.7
	Female	679	85.3
Major	Medical	383	48.1
	Non-medical	413	51.9
Class	Freshman	230	28.9
	Sophomore	226	28.4
	Junior	226	28.4
	Senior	114	14.3
Place of household registration	Rural	510	64.1
	Urban	286	35.9
Level of education	College and below Undergraduate and above	336 460	42.2 57.8
Mean monthly consumption level	< 1000 yuan/month	207	26.0
	1000–2000 yuan/month	505	63.4
	> 2000 yuan/month	84	10.6
Mother’s level of education	Primary school and below	184	23.1
	Junior high school	342	43.0
	High school and technical secondary school	155	19.5
	College and above	115	14.4
Father’s level of education	Primary school and below	114	14.3
	Junior high school	361	45.4
	High school and technical secondary school	189	23.7
	College and above	132	16.6
Cancer history of family members and friends	Yes	158	19.8
	No	638	80.2
Sexual history	Yes	127	16
	No	669	84
Attitudes toward premarital sex	Support	100	12.6
	Neutral	521	65.5
	Oppose	175	22.0
Mother’s participation in cervical cancer screening	Never	184	23.1
	Occasional	116	14.6
	Regular	64	8.0
	Unclear	432	54.3

### Univariate analysis for the knowledge of cervical cancer prevention and treatment among college students in Zhengzhou [(n)%]

Mann–Whitney U testing was carried out by taking the demographic characteristics of 796 college students in Zhengzhou (such as gender, major, grade, place of household registration, level of education, mean monthly consumption level, and parental level of education) as independent variables. A dichotomous variable, namely, the knowledge level of cervical cancer prevention and treatment, was taken as the dependent variable (Table 2). The results showed that there were statistically significant differences in the knowledge level of cervical cancer prevention and treatment between different groups of gender, major, grade, place of household registration, level of education, mean monthly consumption level, mother’s level of education, father’s level of education, cancer history of relatives and friends, sexual history, and attitudes toward premarital sex, and mother’s participation in cervical cancer screening (*P* < 0.05).

**Table 2:** Univariate analysis for the knowledge of cervical cancer prevention and treatment among college students in Zhengzhou [(n)%]

Variable	Group	Poor knowledge	Good knowledge	*Z*	*P*-value
Gender	Male	75 (64.1)	42 (35.9)	-2.542	0.011
	Female	349 (51.4)	330 (48.6)		
Major	Medical	145 (37.9)	238 (62.1)	-8.385	< 0.001
	Non-medical	279 (67.6)	134 (32.4)		
Grade	Freshman	167 (72.6)	63 (27.4)	-8.273	< 0.001
	Sophomore	128 (56.6)	98 (43.4)		
	Junior	91 (40.3)	135 (59.7)		
	Senior	38 (33.3)	76 (66.7)		
Place of household registration	Rural	301 (59.0)	209 (41.0)	-4.342	< 0.001
	Urban	123 (43.0)	163 (57.0)		
Level of education	College and below Undergraduate and above	211 (62.8)	125 (37.2)	-4.603	< 0.001
		213 (46.3)	247 (53.7)		
Mean monthly consumption level	< 1000 yuan/month	117 (56.5)	90 (43.5)	-2.658	0.008
	1000–2000 yuan/month	279 (55.2)	226 (44.8)		
	> 2000 yuan/month	28 (33.3)	56 (66.7)		
Mother’s level of education	Primary school and below	101 (54.9)	83 (45.1)	-3.999	< 0.001
	Junior high school	216 (63.2)	126 (36.8)		
	High school and technical secondary school	63 (40.6)	92 (59.4)		
	College and above	44 (38.3)	71 (61.7)		
Father’s level of education	Primary school and below	60 (52.6)	54 (47.4)	-4.375	< 0.001
	Junior high school	232 (64.3)	129 (35.7)		
	High school and technical secondary school	79 (41.8)	110 (58.2)		
	College and above	53 (40.2)	79 (59.8)		
Cancer history of family members and friends	Yes	66 (41.8)	92 (58.2)	-3.232	0.001
	No	358 (56.1)	280 (43.9)		
Sexual history	Yes	55 (43.3)	72 (56.7)	-2.452	0.014
	No	369 (55.2)	300 (44.8)		
Attitudes toward premarital sex	Support	40 (40.0)	60 (60.0)	-3.788	< 0.001
	Neutral	273 (52.4)	248 (47.6)		
	Oppose	111 (63.4)	64 (36.6)		
Mother’s participation in cervical cancer screening	Never	74 (40.2)	110 (59.8)	-8.190	< 0.001
	Occasional	40 (34.5)	76 (65.5)		
	Regular	14 (21.9)	50 (78.1)		
	Unclear	296 (68.5)	136 (31.5)		

### Logistic regression with multiple covariates for the knowledge of cervical cancer prevention and treatment among college students in Zhengzhou

To identify influencing factors on the knowledge of cervical cancer prevention and treatment after adjusting for confounders among college students in Zhengzhou, logistic regression analysis was carried out with the dichotomous variable, namely, the knowledge level of cervical cancer prevention and treatment, as the dependent variable. The independent variables were the gender, major, grade, and place of household registration, level of education, mean monthly consumption level, mother’s level of education, father’s level of education, cancer history of relatives and friends, sexual history, attitude toward premarital sex, and mother’s participation in cervical cancer screening of college students in Zhengzhou (inclusion criterion α = 0.05, exclusion criterion β = 0.10). The results showed that gender, major, grade, level of education, father’s level of education, attitudes toward premarital sex, and mother’s participation in cervical cancer screening were found to be the independent predictors of the knowledge of cervical cancer prevention and treatment, whereas place of household registration, mean monthly consumption level, mother’s level of education, cancer history of relatives and friends, sexual history were not (Table 3). In addition, we found that female, undergraduate and above, and college students whose mother regularly participated in cervical cancer screening were protective factors for the knowledge of cervical cancer prevention and treatment. Non-medical college students and had opposed attitudes toward premarital sex were risk factors for the knowledge of cervical cancer prevention and treatment. The prediction accuracy of the seven predictors for the dependent variable was 74.4%. The overall model of the regression model was significant (*χ*^*2*^ = 260.659, *P* < 0.001). The results of Hosmer–Lemeshow test showed that the overall fit of the model was good (*χ*^*2*^ = 16.952, *P* = 0.053).

**Table 3:** Logistic regression with multiple covariates for the knowledge of cervical cancer prevention and treatment among college students in Zhengzhou

Variable	*B*	*P-*value	*OR*	95% *CI*
Gender				
Female	-0.956	< 0.001	0.384	(0.230–0.644)
Major				
Non-medical	1.407	< 0.001	4.082	(2.863–5.819)
Grade				
Sophomore	-0.632	0.006	0.531	(0.339–0.832)
Junior	-1.273	< 0.001	0.280	(0.177–0.443)
Senior	-1.484	< 0.001	0.227	(0.125–0.410)
Level of education				
Undergraduate and above	-0.421	0.025	0.656	(0.454–0.949)
Father’s level of education				
Junior high school	0.262	0.296	1.300	(0.794–2.127)
High school and technical secondary school	-0.757	0.008	0.469	(0.268–0.821)
College and above	-0.476	0.132	0.621	(0.334–1.154)
Attitudes toward premarital sex				
Neutral	0.560	0.044	1.750	(1.016–3.017)
Oppose	0.973	0.003	2.646	(1.396–5.013)
Mother’s participation in cervical cancer screening				
Occasional	-0.415	0.145	0.660	(0.378–1.153)
Regular	-0.811	0.034	0.444	(0.210–0.941)
Unclear	0.814	< 0.001	2.258	(1.479–3.447)

*CI* Confidence interval; *n* = 796

### Willingness of college students in Zhengzhou to receive HPV vaccination

A total of 589 (74.0%) college students in Zhengzhou had heard of HPV vaccine, and 92.8% of the students expressed their willingness to be vaccinated or recommend their relatives and friends for vaccination. Only 17.0% of males were willing to be vaccinated or recommend their relatives and friends for vaccination. Among the reasons of willingness to vaccination, 79.0% of the students believed that vaccination was better than no vaccination. There were 40 students who were unwilling to be vaccinated. Regarding the reasons of unwillingness to vaccination, 30.0%, 30.0%, 27.5%, and 17.5% of the students indicated vaccine safety concerns, few vaccinated people around, too high price, and no need to vaccinate, respectively. Table 4 lists the pathways by which college students understood the HPV vaccine.

**Table 4:** Pathways for college students in Zhengzhou to understand HPV vaccine (*n* = 589)

Pathways to understand HPV vaccine	Frequency (n)	Percent (%)
Social welfare promotion	337	57.2
Doctor consultation	141	23.9
Introduction by family or friends	275	46.7
Media (e.g., TV, WeChat, Weibo, magazines)	431	73.2
School	182	30.9
Others	33	0.06

## Discussion

### Knowledge of cervical cancer prevention and treatment among college students in Zhengzhou

In the present study, 87.9% of college students in Zhengzhou indicated that they had heard of cervical cancer. This result was higher than that reported by Chen et al. [7]. A previous study of college students in Yantai, China showed that 73.9% of the students had heard of cervical cancer. A plausible reason is that in recent years, with the improvement of people’s living standards, vaccines have been gradually promoted in China, enabling people to get access to higher-level medical services and more medical knowledge through the media, internet, and community publicity. Therefore, the awareness of cervical cancer prevention, diagnosis, and treatment is gradually enhanced, leading to further awareness of the knowledge about cervical cancer. The proportion of college students with good knowledge of cervical cancer prevention and treatment was 46.7% in the present study. In contrast, in developed countries such as the United States, the correct rate of adolescents’ knowledge about cervical cancer prevention and treatment reached 63.6% [10]. There are still many limitations in popularizing the knowledge of cervical cancer prevention and treatment. Therefore, it is necessary to strengthen the publicity and popularization of cervical cancer prevention and treatment knowledge among college students and enhance their awareness of disease prevention.

### Factors influencing the knowledge of cervical cancer prevention and treatment among college students in Zhengzhou

The results of this study showed that the knowledge of cervical cancer prevention and treatment had significant differences between different groups of gender, major, grade, level of education, father’s level of education, attitudes toward premarital sex, and mother’s participation in cervical cancer screening. High grade, high educational level, and medical major were protective factors for the knowledge of cervical cancer prevention and treatment, consistent with previous studies in China and other countries [11, 12]. This may be due to that the higher the grade and educational level of college students, the more knowledge reserve they can acquire. As for medical students, owing to the particularity of their major, they can understand the knowledge of cervical cancer prevention and treatment through multiple approaches, such as teaching materials, classroom teacher explanations, or hospital practice or traineeship, and thereby pay more attention to their own physical and mental health. In contrast, non-medical students have limited approaches to access the knowledge of cervical cancer. Compared with the group with father’s level of education at primary school and below, college students whose father’s level of education was at high school and technical secondary school showed higher knowledge level of cervical cancer prevention and treatment, coincident with the results of Rani et al. [13]. The higher the parental level of education, the better the family’s financial condition and socioeconomic status, which is conducive to improving the cognition of cervical cancer knowledge.

College students who supported premarital sex could sensitively capture the knowledge of cervical cancer in their life and study. College students whose mother regularly participated in cervical cancer screening appeared to be a protective factor for the knowledge of cervical cancer prevention and treatment, which might be related to the family environment. Mother plays a vital role in the family, and her education, awareness, health beliefs, and practices all influence children’s health awareness and practices. Whether children accept HPV vaccine or not mainly depends on mother’s knowledge of the risks and benefits, rather than children’s subjective willingness [14, 15]. In future research, attention needs be paid to male and non-medical college students with a lower level of education, and various health education modes should be used for intervention to improve their knowledge of the disease.

### Willingness of college students in Zhengzhou to receive HPV vaccination

The main preventive measure for cervical cancer include cervical cancer screening and HPV vaccination. The knowledge of cervical cancer and HPV vaccine directly influences the willingness to get vaccinated [16]. In terms of gender, females showed stronger health knowledge than males among college students. In the early stage, HPV vaccine focused on the prevention of cervical cancer, so females were more inclined to get HPV vaccine. According to the World Health Organization, vaccination is the most cost-effective public health measure to prevent cervical cancer. It is recommended that primary prevention should begin with HPV vaccination in girls aged 9 to 14 years before sexual activity, and some countries have started to vaccinate boys [3]. At present, HPV vaccine has not been given to men in most areas of China, and there are problems such as insufficient supply of HPV vaccine and low vaccination rate. The Chinese Medical Association put forward the principles of HPV vaccination for the general population in the Chinese expert consensus on the clinical application of human papillomavirus vaccine, focusing on how to vaccinate women under special medical conditions in 2021 [6]. In the present study, 74.0% of college students had heard of HPV vaccine, and 92.8% of the students expressed their willingness to get vaccinated or recommend their family members and friends for vaccination. These results indicate that college students have a high willingness to vaccination, despite their relatively poor knowledge of HPV vaccine, which is similar to previous research results in China and other countries [17, 18]. It has been reported that students who have family members and friends with cervical cancer and who are sexually active are more willing to be vaccinated against HPV [7, 13]. College students mainly understand HPV vaccine through the media, social publicity, family members, or friends. Most developed countries have incorporated the HPV vaccine into their national vaccination programs, and the knowledge level of HPV vaccine among college students is higher than in developing countries. Factors such as gender, major, family income, and health literacy can all influence the knowledge level [19–22]. Consequently, society should carry out health education on HPV vaccine for adolescents of appropriate age, while colleges and universities should offer courses related to cervical cancer and HPV vaccine or provide health education lectures, aiming to reduce the obstacles to HPV vaccination and achieve cervical cancer prevention and treatment.

## Conclusions

College students in Zhengzhou had a poor knowledge of cervical cancer prevention and treatment. Their knowledge of cervical cancer prevention and treatment was influenced by different gender, major, grade, level of education, father’s level of education, attitudes toward premarital sex, and mother’s participation in cervical cancer screening. Despite their relatively poor knowledge of HPV vaccine, college students in Zhengzhou expressed their high willingness to get vaccinated. In future research, hospitals and community health service centers should pay attention to diversified health education modes. For people with different characteristics, personalized services can be offered through traditional media, new media, and offline activities to actively promote the publicity of cervical cancer prevention and treatment knowledge and promote the vaccination process.

## Data Availability

The datasets generated and/or analysed during the current study are not publicly available because the broader research project is still ongoing and funded, but are available from the corresponding author on reasonable request.
